# A configurable method for clinical quality measurement through electronic health records based on openEHR and CQL

**DOI:** 10.1186/s12911-022-01763-3

**Published:** 2022-02-10

**Authors:** Mengyang Li, Hailing Cai, Yunlong Zhi, Zehai Fu, Huilong Duan, Xudong Lu

**Affiliations:** 1 College of Biomedical Engineering and Instrument Science, Zhejiang University, Zheda Road 38, Hangzhou, 310027 China; 2 Key Laboratory for Biomedical Engineering, Ministry of Education, Hangzhou, China; 3 School of Industrial Engineering, Eindhoven University of Technology, Eindhoven, The Netherlands

**Keywords:** Clinical quality measure, Clinical quality indicators, Healthcare quality improvement, Data reports, Visualization

## Abstract

**Background:**

One of the primary obstacles to measure clinical quality is the lack of configurable solutions to make computers understand and compute clinical quality indicators. The paper presents a solution that can help clinical staff develop clinical quality measurement more easily and generate the corresponding data reports and visualization by a configurable method based on openEHR and Clinical Quality Language (CQL).

**Methods:**

First, expression logic adopted from CQL was combined with openEHR to express clinical quality indicators. Archetype binding provides the clinical information models used in expression logic, terminology binding makes the medical concepts consistent used in clinical quality artifacts and metadata is regarded as the essential component for sharing and management. Then, a systematic approach was put forward to facilitate the development of clinical quality indicators and the generation of corresponding data reports and visualization. Finally, clinical physicians were invited to test our system and give their opinions.

**Results:**

With the combination of openEHR and CQL, 64 indicators from Centers for Medicare & Medicaid Services (CMS) were expressed for verification and a complicated indicator was shown as an example. 68 indicators from 17 different scenes in the local environment were also expressed and computed in our system. A platform was built to support the development of indicators in a unified way. Also, an execution engine can parse and compute these indicators. Based on a clinical data repository (CDR), indicators were used to generate data reports and visualization and shown in a dashboard.

**Conclusion:**

Our method is capable of expressing clinical quality indicators formally. With the computer-interpretable indicators, a systematic approach can make it more easily to define clinical indicators and generate medical data reports and visualization, and facilitate the adoption of clinical quality measurements.

## Background

Clinical quality measure, measures of processes, experiences and/or outcomes of patient care, observations or treatment that relate to one or more quality aims for health care such as effective, safe, efficient, patient-centered, equitable, and timely care [1], is attracting increasing attention. With specific medical knowledge, clinical quality artifacts should be defined to measure clinical quality. Typically, clinical quality information can be represented in the form of indicators [2]. The Agency for Healthcare Research and Quality (AHRQ) defines quality indicators as “standardized, evidence-based measures of health care quality that can be used with readily available hospital inpatient administrative data to measure and track clinical performance and outcomes” [3].

These clinical quality indicators (CQIs) aim at helping monitor and improve the performance and quality of health care services. By closely monitoring performance and quality, the health workers can identify and take action on things they could do better. It is also important for patients to see for themselves how clinicians are doing, so they can make informed choices about their care. Further-more, it can also make information about clinical quality comparable across different healthcare services providers.

To apply CQIs in clinical practices, many business intelligence (BI) tools are used to develop computable CQIs. Specifically, information technology personnel first communicate with clinical departments or hospital managers to understand the requirements of development and actual use of CQIs. Then they analyze and extract the data elements according to the defined indicators, find the corresponding fields in the constructed data warehouse, and then use these fields to express these indicators. Afterward, they build query statements with BI tools to compute these indicators. Finally, according to the actual usage scenario, they select different styles of report or view components to show the computed results of these indicators. In this process, there are some problems.

First, due to the existence of the knowledge gap, information technology personnel need to communicate deeply with the demand side, such as clinicians, to understand the specific meaning of CQIs in detail. This process needs to be iterated constantly, resulting in huge communication costs. Until the detailed meaning is fully understood, the development and configuration of indicators cannot be carried out. Moreover, BI tools don’t provide any solution to solve the challenge brought by this knowledge gap. Technically, query statements about indicators configured by information technology personnel with BI tools are executed in the data warehouse and the results can be displayed in different components. It is difficult for clinicians to participate in this process of configuration with BI tools. Therefore, in order to solve this communication barrier, we need a solution to separate the technology implementation from the domain knowledge.

Second, BI tools are used in the data warehouse within healthcare institutions. The data models of the data warehouse are different among different healthcare institutions. The indicators configured with BI tools are closely related to the data models of the data warehouse within the institutions. Some indicators need to be shared among different institutions. These shared indicators can not only make the knowledge within indicators reusable but also the consistent results based on the shared indicators can make a comparison among different healthcare institutions. For example, for patients, they can compare these indicators to decide which hospital to go to for treatment. On the other hand, hospitals can improve their performance through the comparison of these indicators. However, the indicators closely bound to the internal data models of healthcare institutions hinder sharing of them. Therefore, we need to define these indicators in a technologically independent way to achieve the goal of sharing. On some platforms, such as CMS in US, National Center for Clinical Laboratories in China. Also, many studies have been conducted about the development of CQIs [4–6]. There are already some indicators expressed in natural language [3, 7, 8] that can be shared in different hospitals. It still leads to inaccurate computation of indicators in practical application because of the fuzzy expression, so that the final results are still incomparable. In general, we need a technology-independent method to express indicators in a formal way.

Although there are some formal indicator expression languages, such as CQL in HL7 whose data model does not separate technical implementation from domain knowledge, the difficulty of communication still exists. OpenEHR is a multi-level modelling method applied to EHR that is future-proof and flexible. It separates data representation from domain content. Technology personnel use reference model to develop software applications while domain experts like clinicians manage and develop domain knowledge. It makes everyone focus on their own areas of responsibility to avoid huge communication costs. In addition, openEHR information models are not bound to the specific technical implementation, and the data elements within them are expressed in a formal way. It avoids the defect of fuzzy expression in natural language and is conducive to the construction of computable indicators.

Considering this, we propose to use CQL, combined with the advantages of the openEHR information model to provide a configurable method for clinical quality measurement through EHR. It can be applied in all types of indicators including process quality, structural quality, and outcome quality because we focus on the general configurable method.

### Clinical Quality Language

Clinical Quality Language (CQL) is an HL7 authoring language standard focusing on the expression of clinical knowledge that can be used within both the Clinical Decision Support (CDS) and Clinical Quality Measurement (CQM) domains. It provides the ability to express logic that is structured enough for processing a query electronically to allow for a more modular, flexible, and robust expression of the logic. It also defines a representation for the expression of clinical knowledge which can help guide the design of clinical quality indicators to make it sharable among healthcare organizations based on several information models, such as the Quality Data Model (QDM) [9] and QUICK [10] logical data model.

In general, CDS and CQM have something in common, like expression logic, also share some requirements. So CQL focuses on the common representation of expression logic that CQM and CDS-specific artifact standards can use. Other specifications, like metadata and data model, are provided separately.

### An example expressed with CQL

An artifact for measuring the clinical quality can be comprised of three involved components [11], including metadata, clinical quality information, and expression logic.

- Metadata: used to describe attributes of the artifact, such as its identifier, version, and state, what healthcare subjects it represents, related artifacts, different languages, authoring, etc.
- Clinical Quality Information: the clinical content used to compute quality indicators involved in this artifact.
- Expression logic: the conditions or rules for reasoning and querying, the core part to compute indicators.

**Fig. 1:**
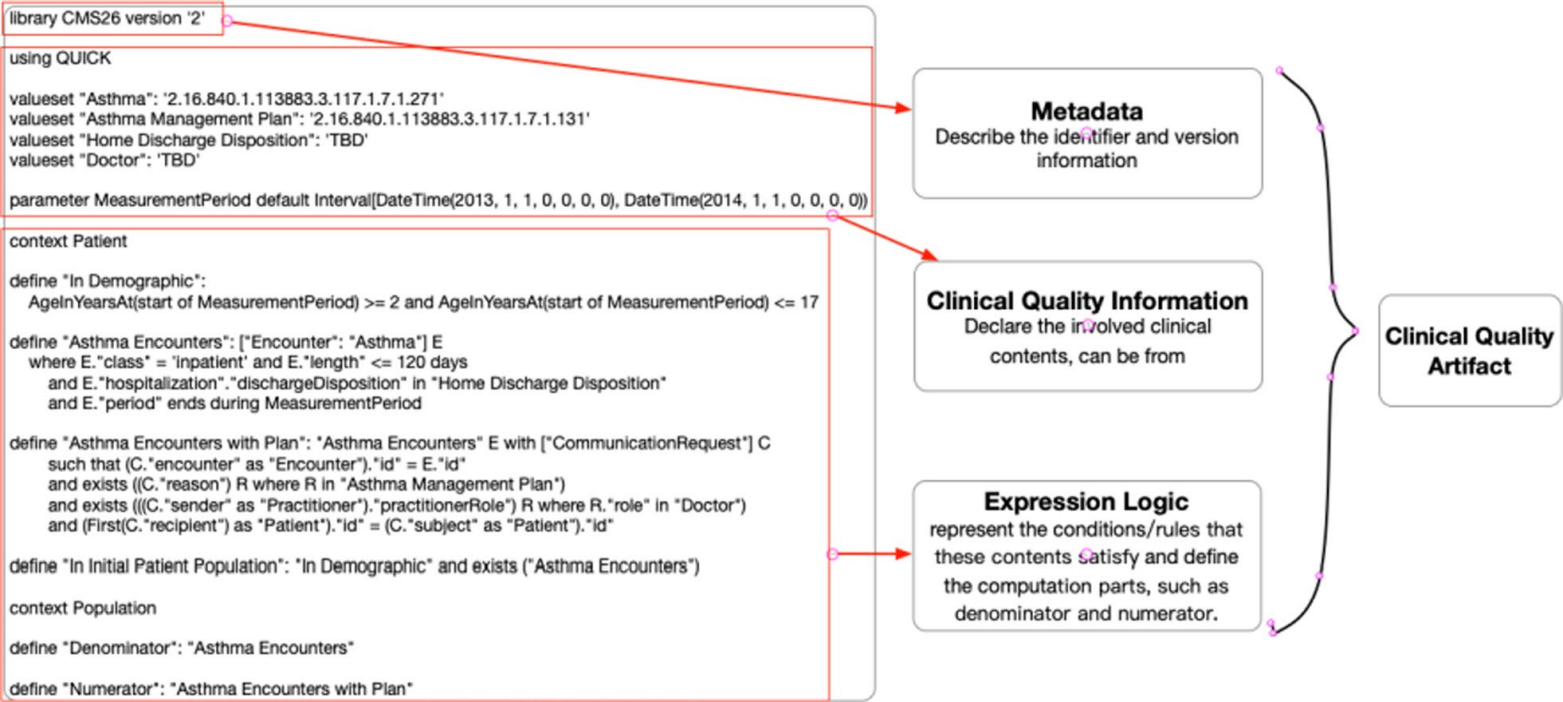
A clinical quality artifact expressed by CQL

A clinical quality indicator expressed in CQL can be helpful to understand the three components intuitively. It is an indicator of an assessment that there is documentation in the medical record that a Home Management Plan of Care (HMPC) document was given to the pediatric asthma patient/caregiver [7]. The detailed representation shows in Fig. 1.

According to this artifact, the first part in line 1 shows that the indicator is defined by CMS with version 2. The next part declared the medical data model used in clinical quality measurement to express related data elements. CQL supports the reference of well-defined data models by ‘using’ keyword. The Encounter class is imported from QUICK data model [10]. Self-defined parameters are also allowed in the declarations. The final part is the expression logic about which conditions to be satisfied to evaluate and compute the indicators.

### OpenEHR specification

As a well-known EHR modeling specification, openEHR [12] is designed to ensure universal interoperability among all forms of electronic data. The approach allows data models to be infinitely flexible and extensible within the constraints of the reference model which can keep up with the development of the clinical knowledge and meet the requirements of the complicated clinical environment [13].

It introduces a multi-level modeling method that separates data representation from domain content [12]. Based on the Reference Model (RM) which represents clinical data structures and types, a series of archetypes can be developed to define the entire attributes related to specific clinical concepts. Different archetypes can be organized into context-specific datasets, templates which are mostly developed and used locally. Only the Reference Model is implemented in software, while clinical information (archetypes and templates) is independent of specific implementations. The decoupling mechanism makes it easier for the domain professionals to produce computable domain content by modeling tools, such as Clinical Knowledge Manager (CKM).

### Other specifications in openEHR

There are also other expression language specifications in openEHR, such as Expression Language (openEHR EL) [14] and Guideline Definition Language (GDL) [15]. OpenEHR EL defines the semantics of all of the elements of computable expressions and are likely to be used in healthcare and life sciences computing where rules and expressions are needed. However, it is still under development so far. And also, no mature and stable grammar and lexical specification is provided. The scope of GDL is to express clinical logic as production rules with discrete GDL rules containing ‘when-then’ statements. It concentrates on the expression and execution of rules and doesn’t support the complicated expression of indicators, such as “count the patients whose APACHE II score not less than 15 within 24 h after entering the ICU”. On the contrary, CQL was designed to overcome the obstacles to point-to-point sharing of clinical knowledge artifacts such as lack of tooling, the complexity of implementation, or insufficient expressivity. It acts as a mature specification and is used in the domains of quality measurement. So, combining expression logic in CQL with openEHR information models is a feasible way to express clinical quality indicators. Clinical physicians can configure these indicators directly by utilizing well-defined information models and complicated expressions from CQL.

### Some BI tools applied in healthcare

Initially, the development of data reports and visualization in healthcare is customized. With the rapid growth of information technology, these requirements can be mainly covered by some business reporting software [16, 17], such as Crystal Report [18], Quickreport [19], etc. The adoption of information technology has greatly improved operating efficiency, clinical quality, and financial effectiveness. Because the power of these BI tools has proved in many industries, mature solutions are also thought to be beneficial to healthcare. At the same time, many large software companies are making efforts in BI tools, and make these tools more and more powerful, like Tableau [20], SAP BusinessObjects [21], IBM Cognos Analytics [22], Power BI developed by Microsoft [23], OBIEE developed by Oracle [24].

Most of them provide rich components, like different kinds of data reports and views, and statistical analysis functions for various problems, like time series analysis. They make it easier to develop and configure indicators to generate complicated and personalized dashboards. Then, seamless integration with other products makes them transparent to develop data reports. For example, Power BI developed by Microsoft can integrate with Excel and SQL Server which makes it acceptable easily for new users who don't have to think too much about the data source. OBIEE developed by Oracle also does the same thing as Microsoft. More than that, some tools also provide data manipulation function which cleans and transforms data from different sources. For example, in QlikView, the introduction of the QlikView data files (QVD files) partly replaces the functions of Extract, Transform, Load (ETL) tools to perform normal data clean operations [25].

The structure of the rest of this paper is as follows. Second section describes the representation of CQIs, including archetype binding, terminology binding, and proposed metadata. And, it presents the experiment carried out in our study. Third section gives the results. Fourth section discusses the contributions of our method, and some relevant issues and limitations. And conclusions are summarized in fifth section.

## Methods

Our configurable method can be divided into two parts, the first part is about using openEHR information models to express CQIs, and the second part is to propose a systematic approach to use and compute these clinical quality indicators, and generate corresponding data reports and visual dashboards.

### Representation of clinical quality indicators

Clinical information and metadata need to be extended and revised while expression logic can be used from CQL directly. There are several difficulties to apply CQL directly to archetype-based context. First, metadata provided by CQL is not enough to archetype-based indicators expression. For example, the status of development and multilingual support are not given explicitly in CQL which are required in archetypes. Second, for data models, different design philosophies lead to different operation approach. In some cases, CQL can support mostly get access to data elements of classes by using “.” operator which is totally different from that in the archetype-based systems by using path-based style. In order to resolve the incompatibility issues and take advantage of the strengths of archetypes, we have proposed three parts:

- Archetype binding: by binding data elements from archetypes to clinical concepts, CQIs can be expressed with openEHR models. For the computation of indicators, information about templates is also included in the binding.
- Terminology binding: Terminology can be used to disambiguate the expression of indicators. To include public terminology sets and private terminologies defined within archetypes, terminology binding mechanism was brought into the extension.
- Metadata: Metadata was designed to help manage indicators easily and share them among different organizations.

#### Archetype binding

**Fig. 2:**
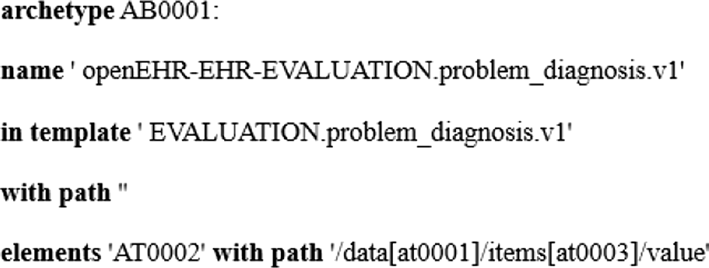
The syntax of archetype binding

The major problem to express indicators in the archetype-based environment is to introduce related clinical concepts. OpenEHR specification is used as the information models to express clinical concepts employing archetype binding mechanism from GDL [15]. The mechanism is a more reasonable way to help design indicators in a unified form. The bound archetypes to the specific indicator can be called archetype binding instances which contain detailed information about archetypes. The content about archetype binding is shown in Table 1. With these defined keywords and functions, the syntax of archetype binding is shown in Fig. 2. There exist many clinical concepts that may come from different archetypes in a clinical quality indicator. A series of local codes are declared to identify different archetypes and clinical concepts, meanwhile, it can make the entire indicator more compact and readable. Before performing the computation of indicators, some criteria need to be satisfied. ‘with predicates’ can help construct such rules to avoid the computation of failure.

**Table 1:** Archetype binding keywords

Keywords	Function
archetype	Define an instance of archetype binding and provide a unique identifier for the instance
name	Define the name of the bound archetype, from where a set of data elements are selected
in template	Define the name of the corresponding template for the specified archetype, optional
with path	Define the archetype’s path in the template by slot mechanism, if no slotted archetypes, an empty string is provided
elements	Define the container including all the bound data elements and provide the corresponding paths
with predicates	Provide a list of predicates (constraints) whose content is a series of expressions

Another issue that needs to be taken into account is the computation in practical applications. An openEHR template consists of a tree of one or more archetypes with corresponding constraints. It is prone to be used locally in application-level systems. So, a kind of representation about the template is also presented. Combining with archetype relational mapping method [26] providing mapping relationships, data about indicators can be fetched from persistent storage databases easily to execute the computation.

#### Terminology binding

A large number of terms exist in the expression of CQIs, such as hypertension, stroke and other medical concepts terms. In the process of the design and expression of indicators, we can use the existing terminology set, such as the SNOMED CT and ICD-10. The HL7 organization defines CodeSystem and ValueSet to promote the use of terms. In the archetype-based environment, openEHR specification uses terminology in a more general way and supports to define terminology set within archetypes to allow the different organizations to use their private terminology in the specified context [27]. In this paper, two ways were provided, one is to use the external terminologies directly and the other is employing defining a set of dictionaries about terms within the archetypes. Terminology binding is required to specify the name of the terminology, the address of the resource and the corresponding terminology encoding content. The keywords used in terminology binding are shown in Table 2.

**Table 2:** Terminology binding keywords

Keywords	Function
term	Indicate the name of the terminology set
uri	Specify the address of the term resource
bind	Identify the code of the term within archetypes
code	Identify the code of the term in the specified terminology set
archetype	Indicate the archetype to which the internal terms belong

**Fig. 3:**
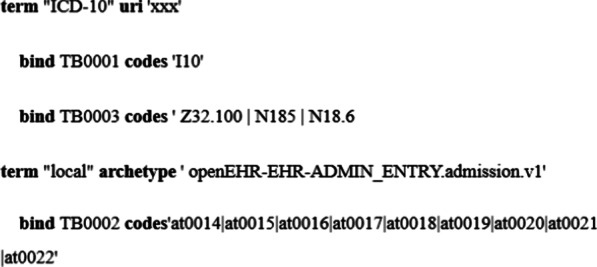
The syntax of the terminology binding

The syntax of the terminology binding is shown in Fig. 3.

For the terms from the external terminology set like ICD-10, we can indicate the name of the terminology set directly. However, for the terms defined within archetypes, the name of the specified archetype should be provided first to restrict the origin of the terms and then we can bind data elements to the corresponding terms.

Some data elements can be bound to one term or several terms in the same terminology set as shown above, such as TB0003 that is a variable parameter is bound to Z32.100 or N185 or N18.6 from ICD-10 which means the parameter TB0003 can be any term from them. The one-to-many terminology binding mechanism can help build the relatively general clinical quality indicators, for example, computing the incidence for different diseases by defining the disease type as variable and binding it to different terms in the same terminology set which makes it possible to avoid building multiple indicators for the similar computation.

Meanwhile, for the use of the single term from external terminology set in indicators, the syntax of its expression is so complicated and redundant. So, to simplify the expression and make it more readable, we design a simple pattern by combining the name of the terminology set and the corresponding code. For example, if the terminology set is ICD-10, the pattern looks like ICD10::[N185].

#### Metadata

The contribution and significance of CQIs are the sharing of knowledge artifacts [28] among different organizations to facilitate the delivery of healthcare services and improve their performance. It requires metadata so that the CQIs can be co-managed and collaborated. To this end, we define a series of metadata, including the name, version, purpose and so on. The details of the metadata are shown in Table 3.

**Table 3:** Metadata keywords

Keywords	Function
indicator	Define the name of the clinical quality indicator
version	Identify the version of the clinical quality indicator
language	The language used in the conceptual description section when the knowledge component is created
translation	Supported translation language, use “|” as separator for multiple translation languages
description	Describe the purpose of the clinical quality indicator
status	The state of the clinical quality indicator, included optionally DRAFT, REVIEWING, PUBLISHED, DEPRECATED
author/time/email	Identify the author, time of creation, and contact information of the author for the clinical quality indicator

These keywords can describe different aspects of an indicator. The status of an indicator provides the development information which reveals the maturity of the indicator to help stakeholders decide to apply it or not. Multiple-language support makes it more convenient to develop once and use multiple times for a diverse language environment.

**Fig. 4:**
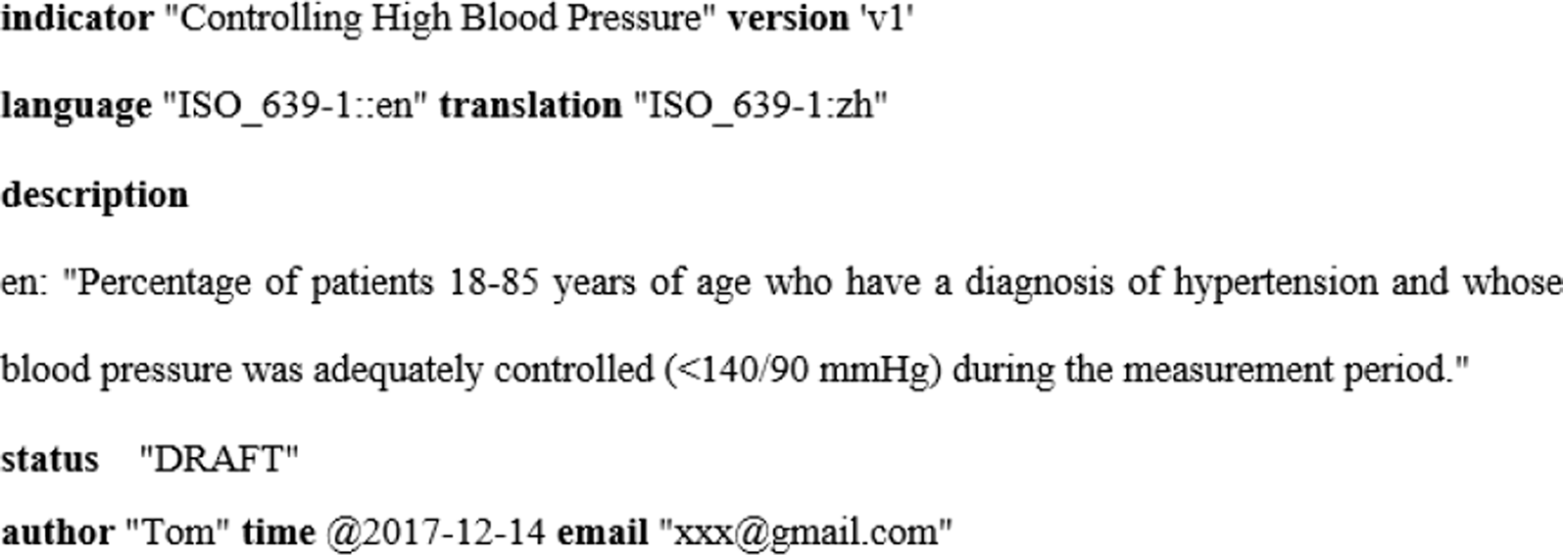
The syntax of the metadata

The syntax of the metadata is shown in Fig. 4.

### Systematic approach for development of CQIs

**Fig. 5:**
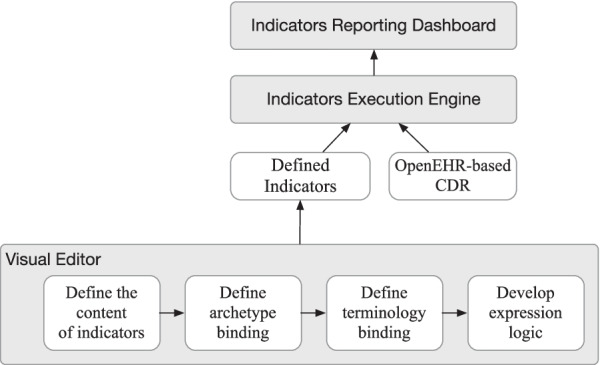
System architecture

To keep different modules independent, our approaches use a modular architecture in which all modules are loosely coupled. In this way, submodules can be developed separately which results in substitutable design by introducing emerging or more advanced methods to allow greater scalability. Besides, this architecture also brought lower development and maintenance costs. It consists of three modules as in Fig. 5.

- an indicator editor as a configurable tool to express these indicators formally,
- parser and executor of indicators to transform them into executable queries,
- reporting system to execute these indicators and fetch data from databases to display in the dashboard.

**Fig. 6:**
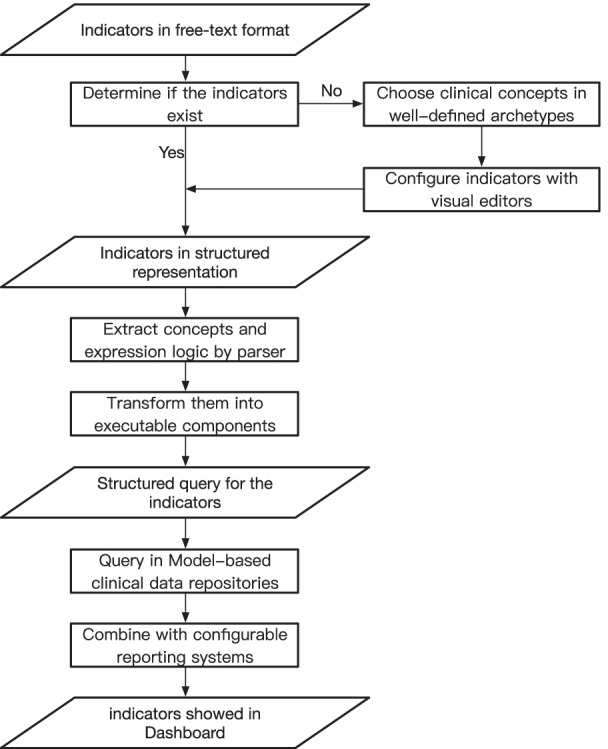
Workflow of proposed approach

According to indicators in free text, the indicator editor provides graphical interfaces to configure them and the tool is integrated with available information repositories to choose concepts from. With the structured representation, parser and executor can be used to extract concepts and expression logic to output executable artifacts to the reporting system. The reporting system fetches data from openEHR-based CDR according to information included in artifacts and generates data reports and visualization. The workflow has been illustrated in Fig. 6.

The indicators in free text format can be referred to from CMS or defined by clinical physicians locally. For the users of the indicators, the first thing for them is to determine if the indicators have existed or not. A management platform was developed to support the retrieval and view of defined indicators. That means you can look them up in this repository. Once you find the approximate one, you can use it directly or just modify a little to go to the next step which improves the work efficiency greatly and avoid time-consuming workload. Complex knowledge involved in indicators and fuzzy expression in natural language make it necessary to provide well-defined information models to choose clinical concepts from. The Clinical Knowledge Manager as a knowledge base can be a source of these concepts. Beyond that, there also exists another platform called Healthcare Modeling Collaboration (HMC) [29] where many well-defined models were developed. It is integrated with our visual indicators editor to provide a major description and representation of clinical knowledge.

**Fig. 7:**
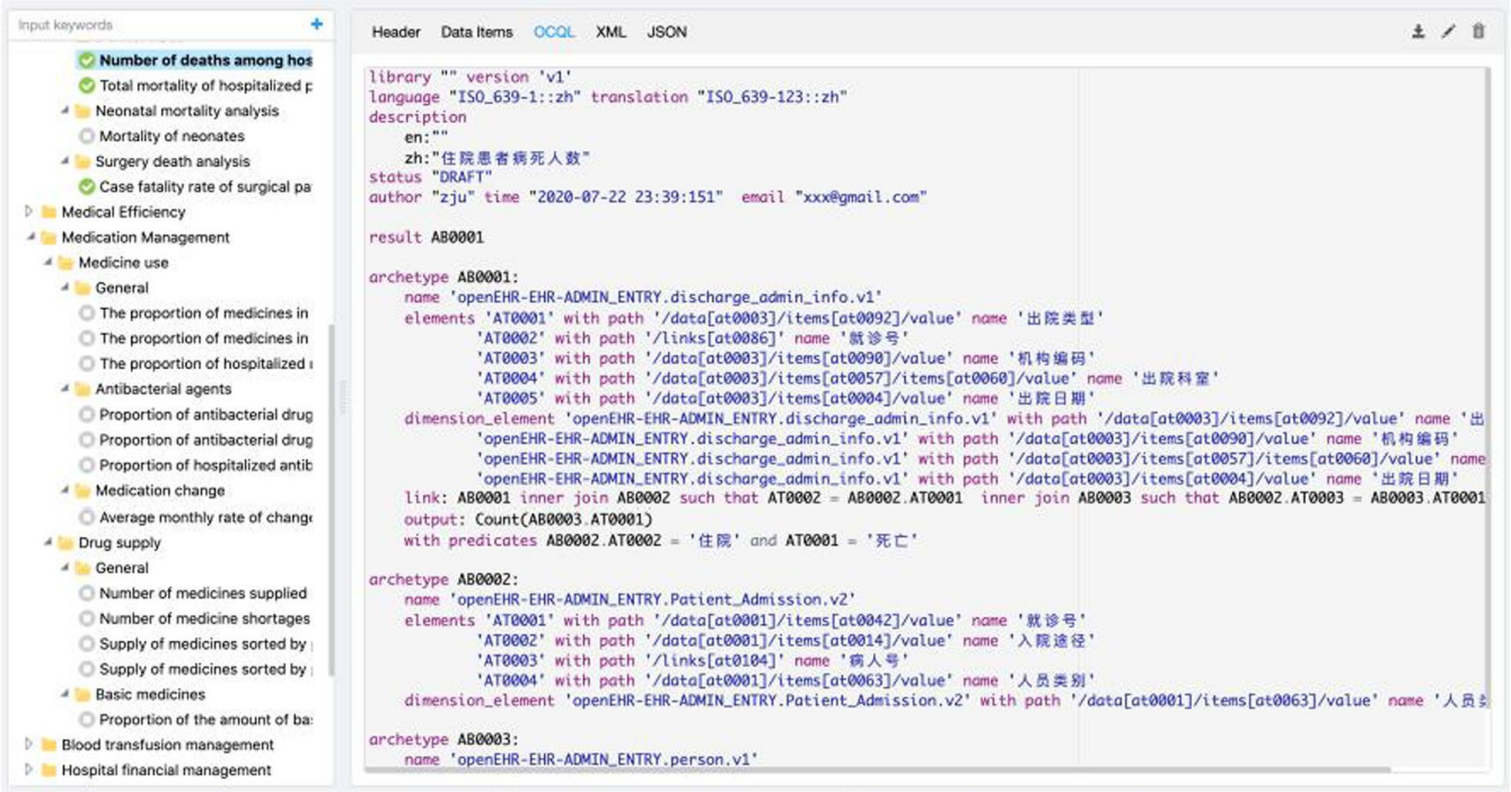
Visual development platform for clinical indicators

The representation of indicators can be the only step that needs domain experts’ participation. To promote the development of indicators, a visual development platform has been published as shown in Fig. 7.

The platform is not just a collection of indicators. It has other functions:

- the retrieval and classification of indicators, well-organized indicators make it convenient to browse and search these indicators;
- status management, for the development of indicators, it points out the different phases of the indicators, including draft, publishment. When the indicators are published, they can be applied in a production environment;
- integration with clinical information models, in this way, predefined models can be used in the interactive interface of definition about indicators;
- serialization for interchangeability, to facilitate the transmission by the network, XML-style representation was supported.

An indicator expressed in a structured format can be built after the steps before. After, ANTLR (ANother Tool for Language Recognition) [30] is used to help build an execution engine. It is a powerful parser generator for reading, processing, executing, or translating structured text or binary files. Based on CQL syntax formal specification [31], a syntax file was formalized and the corresponding parser has been developed with the grammar and lexical specification. The parser was utilized to process and extract information from the indicators in structured text. According to concepts and expression logic included in these indicators, an execution engine was built to query corresponding data. Concretely, the execution engine utilized the parsed information to constitute underlying query statements, like SQL statements. In most cases, indicators are a simple combination of clinical items, such as rate-based indicators. Therefore, only the relevant data need to be fetched and computation can be executed locally rather than in the repositories.

Archetype relational mapping solution [26] was used to build CDR as an experimental environment. The approach is capable of generating relation databases using archetypes and templates for archetype-based EHR systems. The archetypes and templates also can be utilized to support the expression of clinical quality indicators. In this way, the concepts used in indicators were mapped to the databases and lay the foundation for the execution of structured queries. A configurable reporting system makes it possible to generate data reports and charts by ‘drag and drop’ so that it is very convenient and more efficient to monitor these indicators.

From the users’ perspective, based on the openEHR-based CDR and designed clinical indicators, an execution engine facilitates the computation of clinical quality indicators after fetching related data. The entire process is automatic without manual intervention. The computed indicators can be viewed in a reporting dashboard to help make decisions, like sentinel indicators [32].

As a result, the platform acted as a knowledge base about clinical quality indicators. Through the easy-to-use tool, well-organized contents and verified knowledge provided by domain experts, all make it more efficient to measure clinical quality. To evaluate the platform in the actual clinical activities for the definition of clinical quality indicators, we deploy this platform in a hospital to collect the questions/feedback from clinical professionals to help further optimize the platform.

## Experiment

### The representation of clinical quality indicators

We have expressed 64 clinical quality indicators defined and revised by CMS in 2016 successfully. And a complicated indicator was chosen about controlling high blood pressure [33] to describe the detailed process of expressing clinical quality indicators. At the same time, it also can help verify whether our method can express the indicators correctly and unambiguously. The indicator selected here is as shown in Table 4.

**Table 4:** Indicator context for controlling high blood pressure

Items	Detailed description
Measure description	Percentage of patients 18–85 years of age who had a diagnosis of hypertension and whose blood pressure was adequately controlled (< 140/90 mmHg) during the measurement period
Initial patient population	Patients 18–85 years of age who had a diagnosis of essential hypertension within the first six months of the measurement period or any time prior to the measurement period
Denominator statement	Equals initial population
Denominator exclusions	Patients with evidence of end stage renal disease (ESRD), dialysis or renal transplant before or during the measurement period. Also exclude patients with a diagnosis of pregnancy during the measurement period
Numerator statement	Patients whose blood pressure at the most recent visit is adequately controlled (systolic blood pressure < 140 mmHg and diastolic blood pressure < 90 mmHg) during the measurement period
Numerator exclusions	Not applicable
Denominator exceptions	None

First of all, some descriptive contents need to be defined to identify and manage the indicator, such as the name, version, description and author of the indicator. This section corresponds to the metadata management and the content is as follows. And we also predefined a parameter named “MeasurementPeriod” to represent the period of measurement from “2016-01-01 00:00:00” to “2017-01-01 00:00:00”.

Before expressing specific computational logic, data elements used must be determined. These elements and related archetypes from Clinical Knowledge Manager (CKM) [34] are as shown in Table 5.

**Table 5:** Clinical concepts and related archetypes

Archetypes	Clinical concepts
openEHR-EHR-EVALUATION.problem_diagnosis.v1	Problem/Diagnosis name, Date/time clinically recognized, Date/time of resolution
openEHR-DEMOGRAPHIC-PERSON.person-patient.v1 openEHR-DEMOGRAPHIC-CLUSTER.person_birth_data_iso.v1	Birth date
openEHR-EHR-ADMIN_ENTRY.admission.v1	Admit date/time, Admission type
openEHR-EHR-OBSERVATION.imaging_exam.v0	DateTime result issued
openEHR-EHR-OBSERVATION.blood_pressure.v1	Systolic, Diastolic

Then we use these archetypes to declare the archetype binding instances. Meanwhile, there exist many terms in the indicator, such as essential hypertension, nephropathy, pregnancy and so on. The terms can be defined within archetypes or by external reference terminologies according to the terminology binding mechanism. Table 6 shows the terms we used in the indicator.

**Table 6:** Terms used in archetype about diagnosis

Terms	Coded value
Chronic kidney disease	ICD-10: N185
End-stage renal disease	ICD-10: N18.6
Pregnancy	ICD-10: Z32.100
Essential hypertension	ICD-10: I10
Admission type	openEHR-EHR-ADMIN_ENTRY.admission.v1: at0014-at0022

**Fig. 8:**
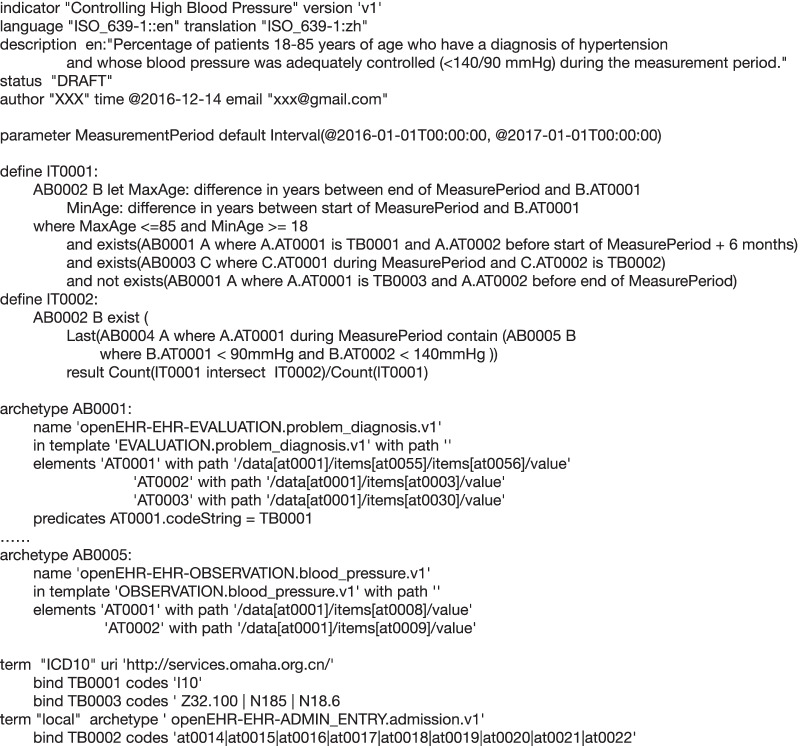
Complete expression for controlling high blood pressure

Based on all of the parts, we express the detailed computation for the indicator. The complete expression is in Fig. 8.

### Usage evaluation from clinical physicians

To evaluate the availability of the proposed method and platform, an experiment was designed and carried out. Our experiment aims at verifying the effect in the process of clinical indicators definition and reports generation. According to the objective, a questionnaire was designed for collecting the opinions and questions. The questionnaire can be divided into two parts, one is to give the pre-defined questions and the other can be collected from clinical professionals directly. This experiment was carried out to show our tool to four clinicians who focus on chronic diseases. First, according to the specific diseases, some archetypes have been prepared to provide convenience for them. Second, indicators for the concrete requirements of clinical departments are presented by the clinicians from their hospital. Third, these clinicians should be trained for a while. The training content consists of a detailed introduction of our platform and the related materials about openEHR information models. Then, they are free to try out this tool independently. During the process, our research assistants just answer questions about the operation on the platform. After the experiment, the participants were required to answer the pre-defined questions. Meanwhile, the follow-up self-directed questions and questions raised during the trial are summarized to help further improve our platform.

## Results

### Representation and computation of indicators

In the experiment, a detailed process was stated to illustrate the representation of indicators with our method. With the presented representation method, in addition to 17 indicators defined in CMS in 2008 [8], and 113 indicators from Hospital Operation, Medical quality and Safety Monitoring Indicators (HMI) proposed by Three Grade general Hospital Evaluation Standard in China in 2016 are also expressed in our platform, including hospital management, surgical complications and so on, the detailed information in Table 7.

**Table 7:** Detailed information about indicators expressed in our representation

Indicators sources	Topics	Numbers of expressed indicators
HMI	Hospital management	29
HMI	Rational use of drugs	21
HMI	Patient safety	15
HMI	Surgical complications	6
HMI	Nosocomial infection	13
HMI	Readmission	13
HMI	Impatient death	16
CMS	Clinical effectiveness	7
CMS	Patient safety	4
CMS	Community/population health	4
CMS	Efficiency and cost reduction	2

The indicators from HMI can be divided into 7 topics, each topic consists of more detailed content, as shown in Table 8.

**Table 8:** Contents about indicators in HMI

Topics	Contents
Hospital management	Basic statistics, such as numbers of different kinds of patients
Rational use of drugs	Include antibiotics use, prescription medication, etc. to carry out statistical analysis and monitoring according to different clinical departments and different types of drugs
Patient safety	Include the safety of surgical patients, newborns, pregnant women
Surgical complications	Postoperative complications according to different diseases and types of surgery
Nosocomial infection	Hospitalization and postoperative infections according to different diseases
Readmission	Rehospitalization, such as Postoperative discharge and readmission because of recurrence of the diseases and other reasons
Impatient death	The number of deaths for hospitalized patients, newborns and pregnant women according to different reasons, etc.

All these indicators were defined because of different purposes and can be used in various scenarios. For example, the government wanted to have an insight into information about medical issues. In general, medical organizations were asked to report to regulated departments about relevant data, such as deaths in hospitals, infection, community and population health. According to the standard to become 3A hospital, these indicators should be more than a specific threshold value.

**Fig. 9:**
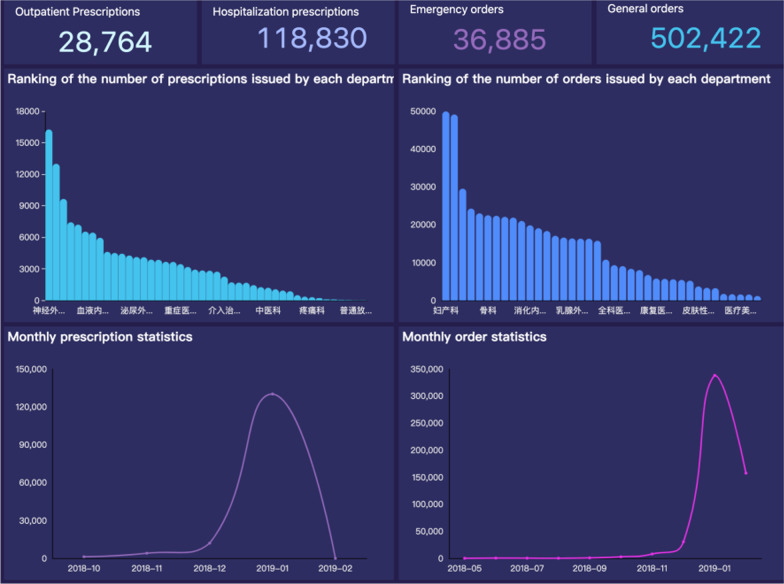
Dashboard for indicators from HMI

Based on the archetype relation mapping persistence solution [26], a CDR was built as the data source for the computation of these defined indicators. 60 templates were used to generate 86 database tables. And a dashboard in the reporting system was in charge of displaying them in Fig. 9.

### The result of usage by clinical physicians

Evaluation of usability has been launched in clinical departments. Four clinicians have been invited to try out our software. And the rating sheet was collected, as shown in Table 9.

**Table 9:** Evaluation of usability from clinicians

Questions	Physician 1	Physician 2	Physician 3	Physician 4
Did you use similar entire configurable tools?	No	No	No	No
How often do you develop indicators? (answers including very few/medium/often)	Very few	Medium	Medium	Very few
Are indicators difficult to develop for you? (yes/no)	Yes	Yes	No	Yes
Do you think the tool is convenient for you compared with methods before? (yes/no)	Yes	Yes	Yes	Yes
Is the tool difficult for you to understand and use?	Need to learn	A little bit	No	Need to learn
Which part is more helpful for defining indicators? (concepts selection/computation/display)	Concepts selection	Concepts selection	Concepts selection	Concepts selection
Is the interactive interface of the tool friendly to use for you? (well enough/need to improve maybe/no)	Maybe	Well enough	Need to improve	Well enough

And some questions and feedback have also been recorded to reflect the limitations of our tool and problems which urgently need to be addressed. Table 10 shows organized and refined content.

**Table 10:** Refined questions and feedback from clinicians

No	Questions & feedback
1	When no available information models, what should I do?
2	Can this tool be automatic processing? I am just responsible for verification
3	There are some similar indicators, can I just modify existing indicators to reuse?
4	The design of the interactive interface is a little bit complicated and I don’t know about openEHR
5	How does the tool combine with our systems and databases, ETL work is boring and time-consuming
6	The tool works pretty good in indicators. I have another problem. When doing medical research, inclusion and exclusion criteria should be satisfied to query relevant patients. Can this tool be helpful?

Accordingly, all physicians admitted it is difficult to develop clinical indicators and thought our tool can be an effective tool to help define them.

From the rating sheet, they showed different views about our tool about usability. Two of them considered it may need some learning costs to master the use of our tool, one of the physicians thought it still is a convenient tool compared with her method before though some knowledge and practices need to be learned carried out. Another physician thought it is easy to use this tool to develop clinical indicators and we found that this physician has a good understanding of information techniques compared with other participants.

On the whole, all of them thought the most important part of the tool is to provide a concept selection function which makes the concepts identified by clinicians and technology personnel. The concept selection task will take a long time according to the feedback from all participants. For example, hypertension can be divided into a primary one and a secondary one. If it is not pointed out and delivered to technique staff clearly, the computation about defined indicators will be wrong. Therefore, they think the concept selection function can play a bridge between them and underlying databases.

Questions and feedback proposed by physicians can be divided into different aspects, such as usability of tools including questions 2, 3, 4, 5, information model development including question 1, other applications including question 6. For the usability of the tool, automatic processing was expected to avoid manual intervention for configuration (It needs to be handled by natural language processing technology.) Information models were a key component for the overall process so that no available models can be considered a problem. One of the clinicians was inspired by our tool to suggest whether it can be applied in medical research for eligibility criteria or not.

## Discussion

This paper presents a configurable solution for clinical quality measurement through EHR based on openEHR and CQL. While the approach provides an effective framework to represent, parse, execute and display indicators in a unified way, there were several encountered challenges and issues.

### Compared with other representations

With the solution presented in the paper, it is helpful to those developers in the openEHR community who works on the development of indicators. Compared to CLIF [35], the method can take advantage of archetypes directly rather than define information models, related terminology and operations. On account of extending from CQL, the method can also support clinical decision support and clinical quality measurement relative to LERM [36]. The limitation of the method is that a relatively small number of indicators were expressed for validation. So, the work in the next stage is to express indicators as much as possible and revise our extension gradually.

### Use of openEHR-based repositories

In our method, the archetype relational mapping persistence solution was used to build CDR. According to the modular architecture, other persistence solutions based openEHR can also be utilized by principle, such as mentioned by Samuel Frade el. [37]. As a multi-level modeling framework, openEHR separates data representation from domain content. In other words, technical personnel should focus on the reference models rather than archetype models. These reference models are the key part to implement persistence solutions. Archetype models are developed and used by domain experts so they should be transparent to underlying technical systems. Clinical concepts included in indicators are defined and chosen from archetype models. In brief, our method was designed to be agnostic to any specific storage implementation based on openEHR.

### Coverage of information models

The major challenge encountered in the process of expression of clinical quality indicators is the absence of corresponding information models. Our method was based on the well-defined models. However, as a result of the complexity of medical knowledge and a huge workload of clinicians, it is more difficult and impossible to cover all the requirements of clinical departments. So, for some users of our solution, defining information models for their needs is necessary to formalize the clinical concepts. From other perspectives, when indicators are expressed in free text, it is hard to understand for information technology personnel. Communication should still need to be done to make sure that the concepts in indicators and databases are consistent. As a consequence, the computation and execution of indicators can be carried out. Much research has been done about information modeling [38–40]. These methods provide experiences and workflow to the modeling work. On the other hand, a visual archetypes editor can help facilitate authoring of openEHR clinical and administrative archetypes which make it lower costs to develop their archetypes.

### Mapping between indicators in free text and structured format

Indicators in free text were proposed by clinical physicians. To handle these indicators by computers, a mapping between natural language representation and structured format should be taken into account. The limitation of our method is that the mapping work still needs to be completed manually by clinical experts. Despite the support of visual editor, there still exists a lot of work to do compared with automatic processing by natural language processing technology. Extra knowledge about models and learning costs still should be known and paid for even if these clinical physicians have already under great pressure of overwhelming work. With the advances of artificial intelligence in natural language processing, named entity recognition and relation extraction have become increasingly robust. Through the adoption of these technologies, clinical concepts can be extracted from the free texts to form structured expressions. In order to accomplish this, unified and rigorous information models and rich terminology support are still essential.

### Limitations about the evaluation in clinical departments

Our evaluation of the tool has some limitations. The first one is the number of users to join in the assessment is few. Although some valuable information has been gathered, a small number of users can result in statistical bias about the conclusion. Therefore, more users can be invited to participate in the assessment to come to a more convincing conclusion. Second, no classification about physicians, different physicians have different levels of involvement, so the impression of the development of clinical quality indicators may be various. Also, this is partly due to a few samples. To address the issues, more users will be invited to try out our tools and classification is also carried out according to familiarity about the development of indicators in the next stage.

### Application in other scenarios

As mentioned in the questions by clinical physicians, for the query of patients in clinical research, our method can be inspired. For the simple criteria, it is easy to express them and execute queries by our tool. The criteria in clinical trials can be more complicated according to an analysis by Weng [41]. And they also have developed a natural language interface to clinical databases [42]. But the ambiguous and vague representations still exist in physicians’ descriptions. For example, “significant medical or psychiatric disorder” and “severe or uncontrolled systemic disease” according to [42], and which raised by physicians, such as “antihypertensive drugs don’t work” and “blood cell count has decreased”. Not all of the problems can be solved by the tool in this paper. To summarize, the simple criteria and the criteria like those which require statistical chart analysis, such as ‘increasing and decreasing’, can be solved by our tool. By defining indicators to describe these statistic variables, the trend and detailed information can be displayed in generated charts.

## Conclusion

Based on the analysis of expression and computation of clinical quality indicators, Expression logic in CQL was combined with openEHR specification. In this way, clinical professionals can define indicators directly which reduces heavy communication costs. The structured clinical indicators make it sharable among different organizations easily. Afterward, a systematic approach was proposed. Based on the developed indicators, an execution engine can compute them and show these indicators in a reporting dashboard. The entire process and configurable tool show that clinical quality indicators can be defined and evaluated quickly and easily. And, the development efficiency of indicators is greatly improved.

## Data Availability

Data sharing is not applicable to this article as no datasets were generated or analyzed during the current study.

## Abbreviations

CQL: Clinical Quality Language
CMS: Centers for Medicare & Medicaid Services
CDR: Clinical data repository
AHRQ: Agency for Healthcare Research and Quality
CQI: Clinical quality indicators
BI: Business intelligence
EHR: Electronic health record
CDS: Clinical decision support
CQM: Clinical quality measurement
QDM: Quality Data Model
HMPC: Home Management Plan of Care
RM: Reference model
CKM: Clinical knowledge manager
EL: Expression language
GDL: Guideline Definition Language
ETL: Extract, transform, load
HMC: Healthcare modeling collaboration
ANTLR: ANother Tool for Language Recognition
HMI: Medical quality and Safety Monitoring Indicators

## References

[1] A quick guide to the clinical quality measures. http://www.cms.gov/Regulations-and-Guidance/Legislation/EHRIncentivePrograms/Downloads/GuidetoCQMs_Remediated_2011.pdf. Accessed 31 July 2020.

[2] Gibberd R, Hancock S, Howley P, Richards K (2004). Using indicators to quantify the potential to improve the quality of health care. Int J Qual Health Care.

[3] AHRQ—Agency for Healthcare Research and Quality. https://qualityindicators.ahrq.gov/. Accessed 31 July 2020.

[4] Voerman GE, Calsbeek H, Maassen IT, Wiegers TA, Braspenning J (2013). A systematic approach towards the development of a set of quality indicators for public reporting in community-based maternity care. Midwifery.

[5] Kötter T, Blozik E, Scherer M (2012). Methods for the guideline-based development of quality indicators—a systematic review. Implement Sci.

[6] McGlynn EA, Asch SM (1998). Developing a clinical performance measure. Am J Prev Med.

[7] CMS26v2 Home Management Plan of Care (HMPC) Document Given to Patient/Caregiver. http://www.nortecehr.com/InPatientContent/CMS26v2.html. Accessed 31 July 2020.

[8] Eligible Professional/Eligible Clinician Ecqms | Ecqi Resource Center. https://ecqi.healthit.gov/eligible-professional-eligible-clinician-ecqms. Accessed 31 July 2020.

[9] Quality Data Model. http://www.healthit.gov/sites/default/files/qdm_4_1_1.pdf. Accessed 31 July 2020.

[10] QUICK Data Model. http://hl7.org/fhir/us/qicore/2018Jan/quick/index.html. Accessed 31 July 2020.

[11] Clinical Quality Language. https://cql.hl7.org/01-introduction.html. Accessed 31 July 2020.

[12] What is openEHR? https://www.openehr.org/about/what_is_openehr. Accessed 31 July 2020.

[13] Thurston LM. Flexible and extensible display of archetyped data: The openEHR presentation challenge. HIC 2006 and HINZ 2006: Proceedings, 28; 2006.

[14] Expression Language. https://specifications.openehr.org/releases/LANG/latest/expression_language.html. Accessed 31 July 2020.

[15] Guideline Definition Language. https://specifications.openehr.org/releases/CDS/latest/GDL.html. Accessed 31 July 2020.

[16] Ferranti JM, Langman MK, Tanaka D, McCall J, Ahmad A (2010). Bridging the gap: leveraging business intelligence tools in support of patient safety and financial effectiveness. J Am Med Inform Assoc.

[17] Using SAS^®^ BI to deliver Ongoing Professional Practice Evaluation (OPPE) At Maine Medical Center. https://www.lexjansen.com/nesug/nesug09/ap/AP05.pdf. Accessed 31 July 2020.

[18] Pixel-Perfect Report Creation, Data Analysis and Report Distribution. https://www.crystalreports.com/. Accessed 31 July 2020.

[19] Quickreport. https://www.quickreport.co.uk/. Accessed 31 July 2020.

[20] Business Intelligence and Analytics Software. https://www.tableau.com/. Accessed 31 July 2020.

[21] SAP BusinessObjects Business Intelligence suite. https://www.sap.com/products/bi-platform.html . Accessed 31 July 2020.

[22] IBM Cognos Analytics. https://www.ibm.com/products/cognos-analytics. Accessed 31 July 2020.

[23] Data Visualization | Microsoft Power BI. https://powerbi.microsoft.com/en-us/. Accessed 31 July 2020.

[24] Business Intelligence (BI). https://www.oracle.com/business-analytics/business-intelligence/. Accessed 31 July 2020.

[25] QVD Files ‒ Qlikview. https://help.qlik.com/en-US/qlikview/April2020/Subsystems/Client/Content/QV_QlikView/QVD_files.htm. Accessed 31 July 2020.

[26] Wang L, Min L, Wang R, Lu X, Duan H. Archetype relational mapping—a practical openEHR persistence solution. BMC Med Inform Decis Mak. 2015;15:88. 10.1186/s12911-015-0212-0. Erratum in: BMC Med Inform Decis Mak. 2016;16:21. PMID: 26541142; PMCID: PMC4636072. 10.1186/s12911-015-0212-0 PMC4636072 26541142

[27] Archetype Definition Language. https://specifications.openehr.org/releases/AM/latest/ADL1.4.html. Accessed 31 July 2020.

[28] Rosenstein AH (1994). Cost-effective health care: tools for improvement. Health Care Manage Rev.

[29] Healthcare Modeling Collaboration. http://hmc.openehr.org.cn/. Accessed 31 July 2020.

[30] ANTLR. https://www.antlr.org/. Accessed 31 July 2020.

[31] CQL syntax formal specification. https://github.com/projecttacoma/clinical_quality_language/blob/master/resources/cql.g4. Accessed 31 July 2020.

[32] Mainz J (2003). Defining and classifying clinical indicators for quality improvement. Int J Qual Health Care.

[33] Controlling High Blood Pressure. https://ecqi.healthit.gov/ecqm/measures/cms165v4. Accessed 6 Nov 2018.

[34] Clinical Knowledge Manager. https://www.openehr.org/ckm/. Accessed 31 July 2020.

[35] Dentler K, ten Teije A, Cornet R, de Keizer N. Towards the automated calculation of clinical quality indicators. In International workshop on knowledge representation for health care. Springer, Berlin, Heidelberg; 2011. p. 51–64.

[36] Medlock S, Opondo D, Eslami S, Askari M, Wierenga P, de Rooij SE, Abu-Hanna A (2011). LERM (Logical Elements Rule Method): a method for assessing and formalizing clinical rules for decision support. Int J Med Inform.

[37] Frade S, Freire SM, Sundvall E, Patriarca-Almeida JH, Cruz-Correia R. Survey of openEHR storage implementations. In: Proceedings of the 26th IEEE international symposium on computer-based medical systems. IEEE; 2013. p. 303–307.

[38] Min L, Tian Q, Lu X, Duan H (2018). Modeling EHR with the openEHR approach: an exploratory study in China. BMC Med Inform Decis Mak.

[39] Maranhao PA, Bacelar-Silva GM, Gonçalves-Ferreira DN, Calhau C, Vieira-Marques P, Alvarenga M, Cruz-Correia RJ. OpenEHR modeling applied to eating disorders in clinical practice: OpenEHR-archetypes in eating disorders. In: 2018 IEEE 31st international symposium on computer-based medical systems (CBMS). IEEE; 2018. p. 36–41.

[40] Kobayashi S, Kume N, Nakahara T, Yoshihara H. Designing clinical concept models for a nationwide electronic health records system for Japan. Eur J Biomed Inform. 2018.

[41] Weng C, Tu SW, Sim I, Richesson R (2010). Formal representation of eligibility criteria: a literature review. J Biomed Inform.

[42] Yuan C, Ryan PB, Ta C, Guo Y, Li Z, Hardin J, Makadia R, Jin P, Shang N, Kang T, Weng C (2019). Criteria2Query: a natural language interface to clinical databases for cohort definition. J Am Med Inform Assoc.

